# Anemia of Inflammation with An Emphasis on Chronic Kidney Disease

**DOI:** 10.3390/nu11102424

**Published:** 2019-10-11

**Authors:** Sajidah Begum, Gladys O. Latunde-Dada

**Affiliations:** 1 Faculty of Life Sciences and Medicine, Henriette Raphael House Guy’s Campus King’s College London, London SE1 1UL, UK; sajidah.begum@kcl.ac.uk; 2 Department of Nutritional Sciences, School of Life Course Sciences, King’s College London. Franklin-Wilkins-Building, 150 Stamford Street, London SE1 9NH, UK

**Keywords:** iron, anemia, kidney, hepcidin, erythropoietin

## Abstract

Iron is vital for a vast variety of cellular processes and its homeostasis is strictly controlled and regulated. Nevertheless, disorders of iron metabolism are diverse and can be caused by insufficiency, overload or iron mal-distribution in tissues. Iron deficiency (ID) progresses to iron-deficiency anemia (IDA) after iron stores are depleted. Inflammation is of diverse etiology in anemia of chronic disease (ACD). It results in serum hypoferremia and tissue hyperferritinemia, which are caused by elevated serum hepcidin levels, and this underlies the onset of functional iron-deficiency anemia. Inflammation is also inhibitory to erythropoietin function and may directly increase hepcidin level, which influences iron metabolism. Consequently, immune responses orchestrate iron metabolism, aggravate iron sequestration and, ultimately, impair the processes of erythropoiesis. Hence, functional iron-deficiency anemia is a risk factor for several ailments, disorders and diseases. Therefore, therapeutic strategies depend on the symptoms, severity, comorbidities and the associated risk factors of anemia. Oral iron supplements can be employed to treat ID and mild anemia particularly, when gastrointestinal intolerance is minimal. Intravenous (IV) iron is the option in moderate and severe anemic conditions, for patients with compromised intestinal integrity, or when oral iron is refractory. Erythropoietin (EPO) is used to treat functional iron deficiency, and blood transfusion is restricted to refractory patients or in life-threatening emergency situations. Despite these interventions, many patients remain anemic and do not respond to conventional treatment approaches. However, various novel therapies are being developed to treat persistent anemia in patients.

## 1. Introduction

Iron is an essential micronutrient required for a number of cellular processes. It is involved in the structure and function of hemoglobin and myoglobin, as well as in the formation of heme enzymes and other iron-containing enzymes of the electron transport chain. Iron is necessary for many biological functions, however, when in excess, toxicity results due to the production of reactive oxygen species and this leads to the malfunctioning of organs [1]. Iron deficiency (ID) describes a condition in which the iron stores in the body are reduced but not sufficiently to limit erythropoiesis. If iron deficiency is severe enough to reduce erythropoiesis, iron-deficiency anemia (IDA) results [2]. In 2016, a systematic analysis for the Global Burden of Disease Study stated that IDA is one of the five leading causes of years lived with disability, particularly in women, and thereby highlighted the prevention and treatment of IDA as a major public health goal [3]. IDA is estimated to affect 1.24 billion people in the world, comprising mostly children and reproductive women, and particularly, in less-developed economies [4]. Iron deficiency (ID) in the absence of anemia has been suggested to be twice the incidence of IDA [5]. Substantive evidence has revealed that both ID and IDA have deleterious consequences on cognition, mental function, work performance, and pregnancy outcomes [6,7]. Furthermore, functional iron deficiency occurs when iron is sequestered in storage organs during inflammation and infections or in situations such as increased erythropoiesis either naturally, due to increased Erythropoietin (EPO) release in response to anemia, or, pharmacologically by erythropoietin-stimulating agents (ESA’s) [8,9].

Anemia describes a state in which there is a reduced erythrocyte count or a reduced level of hemoglobin within erythrocytes [10]. Anemia can be classified in several ways; which can be based on etiological factors, such as nutritional, aplastic, hemorrhagic or hemolytic. However, in clinical practice, classification could be based on the morphology of erythrocytes such as the mean corpuscular volume (MCV). Based on the MCV, anemia can be described as microcytic (MCV< 82 fL), normocytic (MCV = 82–98 fL) or macrocytic (MCV >98 fL). The limitation of this classification is that red cell morphology during hematopoiesis is often not influenced during the early stages of iron deficiency and a class of anemia type could transverse 2 classification groups. Broadly, however, typical examples of microcytic anemia are iron deficiency, thalassemic and sideroblastic anemia. Normocytic anemia includes hemolytic and anemia of chronic disease and folic and vitamin-B12-deficiency anemia are macrocytic.

## 2. Causes of Iron-Deficiency Anemia

Several factors contribute to the development of iron-deficiency anemia and these are presented in a recent review [5]. Physiologically, an increased demand for iron which cannot be met from dietary sources will lead to iron deficiency. This occurs during rapid growth of infants and adolescents, menstrual blood loss, post blood donation and during the first and second trimesters of pregnancy. Nutritionally, inadequate iron intake, malnutrition or poor dietary absorption can lead to iron-deficiency anemia. Pathological causes include decreased absorption and chronic blood loss. Causes of decreased absorption include gastrectomy, bariatric surgery, duodenal bypass, inflammatory bowel disease and atrophic gastritis. Causes of chronic blood loss include bleeding of the gastrointestinal tract (oesophagitis, peptic ulcer, diverticulitis, benign and malignant tumour, hookworm infestation and hemorrhoids), genitourinary system (heavy menses, menorrhagia, intravascular haemolysis (paroxysmal nocturnal haemoglobinuria) and systemic bleeding (trauma, hemorragic telangiectasia and chronic schistosomiasis). Certain classes of drugs have also been implicated in the development of iron-deficiency anemia and these include glucocorticoids, salicylates, non-steroidal anti-inflammatory drugs and proton pump inhibitors. Iron-refractory iron-deficiency anemia is an inherited cause of iron-deficiency anemia. Finally, the availability of iron can be restricted, leading to functional iron deficiency that is associated with anemia of chronic inflammatory conditions [2,5,11,12]. This is a literature review on a few other types of anemia that are associated or concomitant with chronic disease inflammatory conditions. It evaluates the variations in phenotypes, management and discusses the differences in the therapeutic approaches employed.

## 3. Anemia of Inflammation or Anemia of Chronic Disease

Inflammation is an immune response to injury and infection. The inflammatory process causes hypoferremia as an acute-phase response to fight against infection. It involves the secretion of cytokines to regulate iron redistribution, creating hypoferremia that delays pathogen growth, thereby causing the invaders to be engulfed by phagocytes. The orchestrated defense system mounted by the host to fight and fence off pathogens culminates sequentially in tissue iron sequestration, serum iron deficiency, and anemia. This epitomizes the essentiality of life preservation and survival in a competitive hostile environment as normal tissue functions (oxygen delivery) are partially or transiently sacrificed to combat infection [13]. Concomitant with the survival response is the marshalling of the armoury of the erythropoietic drive to override and inhibit both inflammatory and iron-sensing pathways in order to attenuate the downregulation of iron absorption by hepcidin [14,15,16]. Anemia of Chronic Disease (ACD) thus has a multifactorial etiology and has been estimated to afflict over a billion individuals globally [4]. It is prevalent in chronic diseases and disorders, such as heart disease, cancer, inflammatory bowel disease and chronic kidney disease, in which inflammation causes anemia due to increased levels of hepcidin in circulation [4]. The manifestation of the proinflammatory process in a spectrum results in variation in hepcidin levels and the magnitudes of anemia phenotype. ACD, therefore, is caused by a complex interplay of proinflammatory cytokines which induce dysregulation in iron homeostasis, erythroid progenitor cell differentiation, erythropoietin synthesis and red cell longevity, all culminating in the pathogenesis of anemia [17].

Systemic inflammation induced by infection, trauma, dialysis, malignancy or autoimmune disorders activate immune cells to produce cytokines such as Interleukins, (IL) ILI, IL6, IL10, interferon ɣ (IFNɣ) and tumor necrosis factorα (TNFα). Erythropoiesis is impaired by iron restriction, suppression of EPO production and shortened life-span of erythroid progenitors, all culminating in iron-deficiency anemia [17] (Table 1). Thus, ACD is an underlying secondary disorder that is deleterious to the survival of erythrocytes and erythropoiesis.

## 4. Anemia of Cancer

Anemia is prevalent in various types of cancer and iron deficiency accounts for a significant proportion of this comorbidity [18]. The etiology of different tumor types could be multifactorial and complicated by varying underlying factors, but an overriding cause of anemia is chemotherapy-induced. Persistent blood loss, coupled with nutritional deficiencies, culminate in dysregulated iron homeostasis [19]. Features of ID in cancer range from a spectrum of low to high or elevated serum ferritin, a blend of mild absolute ID and a functional ID (FID) gradient. However, as it is a chronic disease that is akin to an inflammatory condition, most cancer patients suffer from FID [19]. It was reported that the prevalence of anemia is about 50% of non-myeloid tumor patients undergoing systemic therapy in a cohort of Spanish hospitals [20]. Moreover, a Europe-wide study that evaluated routine practice in chemotherapy-induced anemia (CIA) management showed that 74% of patients exhibited Hb ≤10 g/dL, including 15% with severe anemia (Hb <8 g/dL). Furthermore 42% of the cancer patients had low-iron levels (ferritin ≤100 ng/mL) [21]. Anemia thus contributes significantly to disease burden and reduced quality of life in cancer patients undergoing therapy. It is, therefore, imperative to treat anemia in the different malignant forms of cancers. Conventional therapeutic approaches include red blood transfusion, the administration of erythropoietin-stimulating agents (ESA), intravenous iron supplementation (IV) and a combination of ESA and IV [22]. While blood transfusion predisposes patients to thromboembolism and increased mortality [22], ESA administration could be refractory in cancer patients. The adverse consequences of IV on oxidative stress and tumorigenesis in heterogeneous cancers are not yet clarified in clinical trials [23]. Recent guidelines and recommendations on the treatment of cancer-related anemia advocate reduction or avoidance of red blood cells (RBC) transfusions, intravenous (IV) alone or a combinatorial use of IV to enhance low-dosage ESA administration [23]. Novel approaches to the treatment of functional anemia that typify chronic diseases, including cancer, are discussed herein later under anemia of chronic kidney disease (ACKD).

## 5. Anemia of Heart Failure

Anemia is prevalent in patients with heart failure (HF), correlates with severity of the disease and is responsible for increased morbidity and mortality in patients [24]. It is characterized by decreased exercise capacity (reduced exercise capacity of 5,6 and worse) by the New York Heart Association (NYHA) functional classification. There are different causes of anemia in HF, arising from the heterogeneous manifestations of the disorder [25]. Anemia caused by absolute iron deficiency in HF may be due to nutritional factors, such as low dietary iron, poor appetite loss, decreased iron absorption due to gastrointestinal blood loss caused by gut inflammation and consequences of iatrogenic agents [26]. However, as it is a chronic disease that is akin to an inflammatory condition, most HF patients suffer from FID [26]. Hemodilution and renal insufficiency are also linked with anemia of HF. Incidence of iron deficiency in chronic HF patients in Europe is about 50%, compared with 61% in Asian populations [27]. A study that analysed about 2000 patients in cohorts in Poland, Spain and the Netherlands reported a prevalence of 32% cases of ID, 12% of IDA and a combination of both as 20% [28]. Anemia thus results significantly in disease burden, increased hospitalizations, and reduced quality of life in HF patients, as well as decreased functional capacity. Conventional therapy for numerous clinical trials is intravenous (IV) iron administration, particularly in patients with symptomatic systolic HF. Intravenous iron infusion has been shown to significantly reduce hospitalizations, improve quality of life, increase exercise capacity and decrease mortality in HF patients. Therapy of symptomatic diastolic HF with IV iron has yet to be clarified or confirmed. Recent guidelines and recommendations on the treatment of HF-related anemia advocate IV iron for symptomatic patients (serum ferritin <100 μg/L, or ferritin between 100–299 μg/L and transferrin saturation (TSAT) <20%) to improve exercise capacity and quality of life [29]. Serum ferritin and TSAT mean values could be variable in HD patients due to confounding factors that are influenced by the magnitude of the symptoms [30]. Such confounders, complexities and the multifactorial nature of iron metabolism dysregulation [31] possibly account for the unreliability of serum hepcidin as a clinical marker of iron status in HD patients [32]. The choice of IV administration of iron compounds varies in different countries.

## 6. Anemia of Surgery

Anemia is prevalent in patients undergoing major surgery and poses an additional independently modifiable risk to patients undergoing blood transfusion [33]. Anemia can range from 7–35% in orthopaedic surgery patients and is associated with high morbidity and mortality [34]. Iron deficiency due to increased requirements, reduced absorption, increased lysis and losses of red blood cells are the main causes of preoperative anemia. Moreover, anemia prior to surgery coupled with increased lysis and losses of red blood cells, iron-sequestration and restricted erythropoiesis lead to over 80% prevalence in postoperative anemia [35]. Perioperative anemia manifests mostly as absolute and functional iron-deficiency anemia that are respectively characterized by scarcity and sequestration of iron in tissues [36]. A chronic inflammatory condition accentuates iron sequestration and FID in the patients. Of note also is that hematinic deficiencies lead insidiously to latent iron deficiency without anemia. A large study of hospital patients of diverse surgical procedures (cardiac, gynecological, colorectal/liver cancer resection) reported the overall prevalence of anemia as 36%. In the anemic patients, 62% had absolute iron deficiency, while FID was 10% [37]. Women accounted for more than twice in number in the cohort. Perioperative and postoperative anemia thus contribute significantly to disease burden and reduced quality of life, the magnitude of which varies with the different disorders. Conventional therapeutic approaches advocate the diagnosis and treatment of anemia before any surgical procedure. Iron deficiency without anemia needs to be treated to replenish iron stores for preoperative requirements and postoperative anemia challenge [38]. Preoperative oral iron could be prescribed for mild-to-moderate anemia patients that are tolerant and do not suffer from adverse gastrointestinal consequences. IV iron supplementation, preferably as a large single dose, is the therapy guideline in moderate to severe postoperative anemic patients. However, a combination of IV and ESA is recommended only in severe anemia that is refractive and resistant over a long period of time.

Red blood cell transfusion (RBCT) may be an acute, inevitable option to correct severe anemia in critically physiologically drained patients after surgery. Guidelines and recommendations for RBCT are restrictive because of the attendant risk of infection, thromboembolic events, high morbidity and mortality in the patients [39,40]. Patient blood management (PBM) that involves evaluation of the hematological status of patients will not only prevent preoperative anemia, but also reduce intraoperative transfusion risk and postoperative complications [41]. Recent guidelines and recommendations advocate a preoperative Hb <13 g/dL to be considered as suboptimal in both men and women and treated before any major surgical procedure [38].

## 7. Anemia of Inflammatory Bowel Disease (IBD)

Anemia is a common comorbidity of inflammatory bowel disease (IBD). The aetiology of IBD is multifactorial and the pathogenesis is complicated by varying underlying factors, such as genetic predisposition, immune dysregulation, loss of mucosal integrity and intestinal microbial composition. This results in a spectrum of chronic relapsing inflammatory disorders that are characterized by ulceration and bleeding of the mucosal epithelium. An inflammatory condition in IBD elevates hepcidin levels in circulation, hence functional iron deficiency ensues; however, chronic intestinal bleeding results also in absolute iron deficiency. Consequently, negative regulators (erythroferrone, growth differentiation factor 15 (GDF-15), platelet-derived growth factor-BB (PDGF-BB) and/or hypoxia-inducible factors (HIFs)) of hepcidin expression dominate to enhance iron absorption from the gastrointestinal tract [42]. Anemia in IBD could arise from several factors that include intestinal blood loss, medications and reduced iron absorption that is also a consequence of reduced appetite during active flaring episodes of the disorder. Moreover, vitamin deficiencies such as those of folate and vitamin B12 are common due to decreased absorption from abnormal duodenum. Absolute iron deficiency caused by diminished absorption and depleted iron stores leads to anemia. Other extenuating consequences of pathological conditions promote acute-phase reactants and cytokines that impair erythroid differentiation and proliferation [43]. A retrospective cohort study, of patients diagnosed with Crohn’s disease and ulcerative colitis during the period 1963–2010 was randomly selected from the population-based IBD cohort of Örebro University Hospital in Sweden and revealed a mean annual incidence rate of anemia as 15.9 per 100 person-years and a prevalence of 22.6%. Of this, anemia was 19.3 per 100 person-years and the prevalence was 28.7% in Crohn’s compared with 12.9% and 16.5%, respectively, for ulcerative colitis [44]. In earlier reports, however, anemia incidence correlated with the disease activity rather than type, although anemia was higher in women [45]. Apart from the consequences of anemia, such as fatigue, headache, dizziness, shortness of breath, or tachycardia, anemia exerts a significant impact on the quality of life of IBD patients. Disease burden caused by abdominal pain or diarrhoea is compounded by persistently debilitating chronic fatigue that is due to anemia [46].

Conventional management and therapeutic approaches recommend anemia screening every 3 months for outpatients with active disease and 6 to 12 months for those in the mild or remission state [47,48]. The thresholds for diagnosis according to World Health Organization (WHO) guideline in adult males and non-pregnant women, stipulate haemoglobin (Hb) of <13.0 g/dL, <12.0 g/dL, and <11.0 g/dL in pregnant women [48]. Specifically, to screen for anemia in IBD patients [49] TfS <20% and a serum ferritin concentration <30 g/L (with a serum CRP level within the normal range or a ferritin concentration of less than 100 g/L with an elevated serum CRP level) are specified. Recommendations for anemia management in IBD patient care are currently conflicting and remain, thereby, an ongoing process because of limited evidence from human studies. However, recommendations for oral iron therapy should be limited to IBD patients with mild anemia and with due considerations given to doses, duration and the types of iron compounds used. This is with the objective to maximize efficiency and efficacy while minimizing side effects. In IBD patients with moderate to severe anemia, oral iron causes gastrointestinal disturbances and is refractory, then, intravenous (IV) iron is the preferred recommendation [50,51]. Inhibition of iron absorption by hepcidin-induced inflammation is by-passed by IV iron to replenish iron stores and replete Hb levels in the patients. ESA, in combination with IV, is prescribed for FID in IBD patients and blood transfusion is an option as an acute measure only in critically anemic patients [50]. Iron therapy and treatment of the other symptoms of IBD will culminate in the reduction of disease burden to improve the quality of life of the patients.

## 8. Anemia of Rheumatoid Arthritis (RA)

Patients with rheumatoid arthritis (RA) may display IDA and anemia of chronic disease (ACD). IDA could be due to gastrointestinal bleeding, gynaecological blood loss, or urinary bleeding or chemotherapy-induced [51]. As inflammation is a chronic condition in RA, FID is common. ACD in RA arises from several factors, including ineffective erythropoiesis inflammatory markers (e.g., IL-6 and TNF-α), and disordered iron metabolism. Functional iron deficiency in ACD can be due to overexpression of iron-regulatory hormone, hepcidin, leading to sequestration into storage sites from circulation, resulting in hypoferrinemia and iron-restricted erythropoiesis. Although decreased serum hepcidin levels were reported to correlate with the reduction of disease activity [52], this observation was not evident in other studies [53,54]. Hepcidin could be suppressed, independently of inflammation. Therefore, the use of hepcidin as a diagnostic tool in the routine clinical management of this disease still requires further investigation. Prevalence of anemia in RA has been reported to range from 64–70%, while ACD was observed in 50–60% of patients [55,56]. RA patients have been reported to have both physical disability and increased mortality [57,58]. Relief in swollen, painful, tender joints, pain, muscle strength, and energy levels symptoms has been reported because of the resolution of anemia in RA patients [58].

Since anemia in RA is multifactorial and often associated with other malignancies, it is important to diagnose the nature and the types of anemia in order to apply the most appropriate treatment regimen. The safety and the use of EPO in the treatment of ACD in RA are controversial [59]. However, regarding the vital role of IL-6 in ACD, a recent study reported a significant increase in Hb and Hct levels after IL-6 receptor inhibitor tocilizumab (TCZ) therapy in anemic and non-anemic patients with rheumatoid arthritis, compared with other biologic and non-biologic disease-modifying antirheumatic drugs (DMARDs) [60].

## 9. Anemia of Chronic Kidney Disease

**Figure 1:**
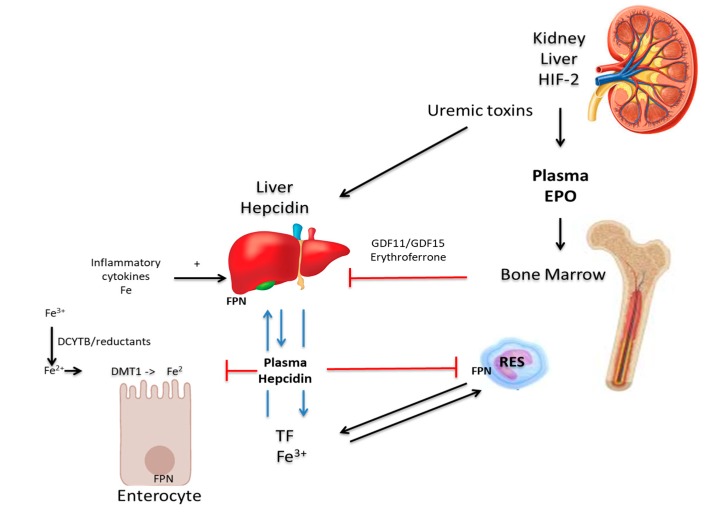
Iron metabolism and the mechanisms of renal anemia. In the enterocyte, duodenal cytochrome b (DCYTB) and other dietary reducing agents reduce ferric iron (Fe^3+^) to its ferrous (Fe^2+^) state, via the divalent metal transporter 1 (DMT1). Iron efflux into the circulation occurs via hepcidin-regulated ferroportin (FPN). In blood, iron is transported bound to transferrin (TF) to the liver, cells of the reticulo-endothelial system (RES) and to other tissues and organs. Inflammatory cytokines suppress erythropoiesis in the bone marrow and stimulate hepcidin production in the liver, which influences iron absorption and efflux negatively. Decreased GDF11/GDF15 or erythroferrone leads to increased hepcidin production. Uremic toxins enhance hepcidin expression and modulate the EPO level via Hif-2α, which also induces the transcription of DCYTB, DMT1, FPN, and TF [72].

Chronic kidney disease (CKD) is a condition in which renal function deteriorates over time as the glomerular filtration rate (GFR) declines progressively. Anemia in CKD is of clinical concern as it predisposes patients to cardiovascular disease, and is associated with poor quality of life, increased hospitalizations, impaired cognition and mortality [61]. Anemia is a consequence of chronic kidney disease (CKD), principally because of the depreciation of and reduced synthesis of erythropoietin. Hence Glomerular Filtration Rate (GFR) is a predictor of anemia in patients with CKD [62]. The progression of anemia leads to several debilitating symptoms, such as lethargy, muscle fatigue, and deterioration of renal function. These culminate ultimately in a high prevalence of cardiovascular diseases, such as left ventricular hypertrophy and heart failure, which account for a significant number of mortalities in patients with CKD [63,64]. However, the etiology of anemia in CKD is multifactorial. EPO is an anti-apoptotic hormone, produced by the kidneys, which promotes the survival, proliferation and differentiation of erythrocyte precursors [65,66]. As CKD progresses, renal mass declines, which reduces EPO production and EPO-deficiency results [66]. The expression of EPO is regulated by the transcription factor, HIF-2α. EPO is produced by peritubular interstitial fibroblasts in the renal cortex and outer medulla and not by the renal tubular epithelial cells or peritubular endothelial cells [67,68], as previously presumed. The regulation of EPO production is by HIF 2α and is modulated by oxygen pressure in the cells and tissues [69,70]. Ablation of HIF-2α, and not HIF-1α, was shown to cause anemia that was restored by recombinant EPO [71]. The regulation of erythropoietin by HIF-2α is also confirmed by increased erythropoietin levels and the ensuing erythrocytosis when the HIF-2α translation is de-repressed in iron regulatory protein 1 (IRP1) knockout mice [72,73,74,75]. Under normoxia, HIF-2α is hydroxylated by O_2_-and iron-dependent HIF prolyl-4-hydroxylases (HIF-PHD) and targeted for proteasomal degradation in a E3-ligase complex. However, under hypoxic conditions, HIF-2α is stabilized and is no longer degraded but translocated to the nucleus where it forms a heterodimer with HIF-β or the aryl hydrocarbon receptor nuclear translocator (ARNT). HIF-2α/β heterodimers, together with transcriptional coactivators, such as CREB-binding protein (CBP) and p300, bind to consensus elements in the 5’ or 3’ regions of the gene for the kidney or liver, respectively, to initiate and increase EPO transcription. Factors such as iron chelators, nitric oxide, ROS or CoCl_2_ inhibit HIF-PHDs association (increased HIF-2α), which culminates to increase EPO transcription and production [72]. Conversely, excess iron was shown to decrease levels of HIF 2α and EPO expression in an erythropoietin-deficient mouse model [76]. Furthermore, kynurenine, a product of L-tryptophan catabolism is increased in anemia of inflammation and in CKD [77]. Kynurenine activates ARNT and competes with HIF-2α to prevent its binding to HIF-β, thereby decreasing EPO production. Similarly, in CKD, a uremic toxin, indoxyl-sulfate, apart from simulating hepcidin expression [78], may also activate ARNT to suppress EPO production [79]. Consequently, an elevated hepcidin level is caused by a medley of interacting factors, such as inflammation, excess iron, decreased EPO/erythropoiesis or metabolites or products of certain processes or pathways of systemic metabolism (Figure 1). The mechanisms by which each player directly influences EPO production are still not yet clearly defined.

In the advanced stages of CKD, regular hemodialysis contributes to absolute iron deficiency. Blood is lost during the hemodialysis process in the tubing and the apparatus and also, through the numerous blood samples taken from the patient [80,81]. Under normal physiological conditions, macrophages engulf senescent erythrocytes and recycle the iron incorporated in hemoglobin [82]. During such blood losses, this opportunity for iron recycling is lost [77,83]. After blood loss, in healthy individuals, EPO aids in the absorption of iron, but this is reduced in CKD patients as they suffer from EPO deficiency as their condition deteriorates. Therefore, in CKD, there is difficulty in replenishing iron stores and consequently, erythropoiesis is limited [84].

### Hepcidin Expression and Function in CKD Patients

Iron availability is the rate-limiting step in the maturation of erythroblasts into erythrocytes [9]. EPO increases the synthesis of erythrocytes in the bone marrow and this leads to a depletion of iron stores and the reduced availability of iron contributes to anemia in CKD [66,85]. Replenishing these iron stores in CKD is also more difficult than in healthy subjects, thereby exacerbating the problem [86].

Furthermore, the reduced glomerular filtration rate in CKD results in impaired renal clearance of hepcidin. Dialysis reduces hepcidin level; however, this rapidly rises again in the interval between dialysis sessions [85]. Mobilization of iron from hepatocyte and reticuloendothelial stores is restricted, leading to absolute iron deficiency due to reduced intestinal absorption of iron [87]. This impairs erythropoiesis, which is iron-dependent and contributes to anemia. CKD patients have a greater predisposition to infection as long-term hemodialysis exposes the patients repeatedly to pathogens in the environment [88]. As previously discussed, this inflammatory state promotes increased hepcidin levels, which contributes to impaired iron absorption and mobilisation [89,90]. Hepcidin produces these effects by downregulating FPN function and iron efflux into the blood. [9,87,91].

Hepcidin expression is suppressed by erythropoiesis to meet iron demand to support the process and EPO has been reported to have a direct effect [16,89,92], possibly in conjunction with co-factors, such as twisted gastrulation protein homolog 1 (TWSG 1), growth differentiation factor 15 (GDF15), GDF11, and erythroferrone (ERFE). The functions of TWSG-, GDF11 and GDF15 in the inhibition of erythropoiesis are still controversial. However, erythroblasts synthesise ERFE upon stimulation by EPO and this inhibits the expression of the hepcidin gene [84,93], particularly under stress. As CKD progresses, reduced production of EPO results in dwindling erythrocyte production and consequently, decreased erythroferrone production. This, in turn, leads to increased hepcidin expression, which reduces iron absorption and decreases iron mobilisation from the stores [93,94]. Reduced iron levels limit erythrocyte maturation and exacerbate anemia of CKD even further.

The regulation of hepcidin induction at the cellular level and in the liver is both intricate and complex and involves membrane-bound iron sensors that include the transferrin receptors (TfR) 1 and 2, HFE, and hemojuvelin (HJV). The signal for hepcidin expression is initiated by bone morphogenic protein (BMP) ligands using the glycosylphosphatidylinositol-anchored membrane protein, HJV, as a coreceptor that binds to Type I and Type II BMP serine threonine kinase receptors. This induces a cascade of activation and phosphorylation of the receptors that channel downstream to Suppressor of Mothers Against Decapentaplegic (SMAD) proteins involved in signalling during hepcidin expression. A detailed description of the triggers that regulate the hepcidin expression process is reviewed elsewhere [14,18]. In summary, hepcidin expression is regulated by the BMP6-HJV-SMAD and IL-6-STAT3 signaling cascade. BMP6 binds the BMP receptors and HJV coreceptor, which causes the phosphorylation of SMAD1/5/8. Phosphorylated SMAD proteins associate with SMAD4 and these complexes traverse the nuclear membrane to bind to the promoter region of the hepcidin gene to induce hepcidin expression. The inflammatory stimulus, IL-6, binds the IL-6 receptor and activates Janus kinase 2 (JAK2), which phosphorylates Signal Transducer and Activator of Transcription 3 (STAT3). Phosphorylated STAT3 translocates into the hepatocyte nucleus to bind the STAT3 responsive element at the promoter region of the hepcidin gene to induce hepcidin expression.

## 10. Treatment of Anemia in CKD

To treat anemia in CKD, it is necessary to enhance the synthesis of erythrocytes, as well as ensure the maintenance of adequate levels of iron for hemoglobin formation [88,95]. The National Institute for Health and Care Excellence (NICE) recommends the use of either iron or erythropoiesis-stimulating agents, or both in combination, for the treatment of anemia of CKD [96]. This is aimed at addressing both absolute and functional iron deficiency that lead to restricted iron access and EPO deficiency [97]. As deficiency in EPO is a major contributory factor to anemia in CKD, recombinant human erythropoietin (rHuEPO), such as epoetin alpha and epoetin beta, are used in the treatment of anemia in CKD patients [98]. This initial therapy was brought into clinical practice in the 1980’s and has been found to successfully treat the signs and symptoms of anemic CKD, such as fatigue, weakness and headaches [99]. In addition, these patients also required less frequent blood transfusions, a further benefit from the use of ESAs in anemic CKD [99]. However, large randomized control studies including the Normal Hematocrit Study (NHCT), the Correction of Haemoglobin and Outcomes in Renal Insufficiency (CHOIR) trial, the Cardiovascular Risk Reduction by Early Anemia Treatment (CREATE) trial and the Trial to Reduce Cardiovascular Events with Aranesp Therapy (TREAT), which highlighted the potential harm of high-dose ESA therapy, have challenged these therapeutic claims from utilising ESAs [100]. It has been emphasized that the complete correction of anemia, and indeed raising hemoglobin levels above 11 g/dL was associated with adverse outcomes. These include an increased risk of stroke, cardiovascular incidents, rapid malignant progression in cancer patients and increased mortality in other patients [61]. Moreover, pure red blood cell aplasia can be induced in rare instances through the use of ESAs, which, in turn, promotes severe anemia and results in the patient becoming transfusion-dependent [66]. It was proposed that these adverse outcomes were due to the very high doses of ESA that are administered [61]. Such high doses of ESA are often prescribed to patients that are hyporesponsive to ESA therapy to correct their hemoglobin deficits and higher doses are provided to attain target hemoglobin levels. The results from randomized controlled trials have subsequently influenced KDIGO guidelines, which recommend that non-dialyzed CKD patients are not administered ESA if haemoglobin levels are above 10 g/dL. CKD patients on dialysis should receive ESA therapy when hemoglobin levels lie between 9 g/dL and 10 g/dL. In all adult patients, ESA therapy should be used to maintain haemoglobin levels no higher than 11.5 g/dL [100].

Evidence from large randomized control studies highlighting the negative health effects of high- dose ESA administration was the reason for advocating the use of iron therapy as an adjunct to ESAs. Consequently, iron and lower doses of ESA are currently prescribed to preclude the adverse outcomes associated with high-dose ESA administration [66]. Moreover, as ESA therapy acts to increase erythropoiesis, this results in the depletion of the iron pool, causing a relative iron deficiency for which iron supplementation is recommended as a preventive measure [61]. Inflammation inhibits erythropoiesis, which influences erythropoietin (EPO) hyporesponsiveness [101] and decreases the systemic circulation of iron levels by the production of hepcidin [102,103]. Inflammation in CKD, apart from causing decreases in iron availability via elevated hepcidin levels, also directly aggravates anemia by suppressing EPO production [104]. Inflammation also decreases the enhancing effect of EPO on erythropoiesis [105].

## 11. Iron Supplementation for the Treatment of Anemia of CKD

CKD patients, as previously explained, suffer from increased blood loss and reduced intestinal absorption of dietary iron and thus, iron supplementation is important to prevent absolute iron deficiency. Iron supplementation may be administered through the oral or IV route, nevertheless, both routes have advantages and disadvantages.

Oral administration using ferrous sulphate is adequate for moderate anemia, and the advantages include its relative low cost [99]. However, the side effects include constipation, nausea and abdominal discomfort, as well as reduced patient compliance [98,106]. Additionally, intestinal iron absorption can be impaired in CKD and the efficacy of oral iron can be variable [99]. Incidentally, sucrosomial iron (SI), a newly developed oral iron preparation, in a randomized trial in CKD patients, has been shown to be comparable to IV iron gluconate in elevating hemoglobin levels [107]. The future of oral iron therapy may involve dietary supplementation with nanoparticles. Nanoparticulate tartrate-modified Fe (III) poly oxo-hydroxide (Nana Fe (III)) has also been shown to be absorbed by a DMT1-independent mechanism for replenishing hemoglobin levels in mice without the side effects associated with oral therapy [108]). The dosage of oral and IV iron in CKD patients are dependent on the presence or absence of inflammatory status of the gut. In Europe and USA, higher doses of IV iron have been used in dialysis patients because of higher inflammation status than in those of Japan. Low doses of IV iron or oral iron have been effective in the Japanese dialysis patients with the same efficacy because inflammation is minimal [109]. Although compared with Western countries, the Japanese guidelines for prescription of IV iron in dialysis patients are more conservative, the outcomes, nevertheless, are as good or better than their American counterparts [110]. Given the potential safety issues with aggressive IV iron treatment, and lack of well-powered studies to examine safety, a more conservative approach to iron therapy should be considered in the US [111].

Intravenous administration is highly efficient at replenishing iron stores, enhancing erythropoiesis and reducing the required ESA dose. This practice is advantageous as high doses of ESA therapy have been associated with negative clinical outcomes [112]. The pitfalls of IV therapy, however, include the invasive method of delivery and increased infection risk [99]. Results from studies of IV iron use and infection have highlighted conflicting results [113,114]. Data from observational, laboratory and animal studies have also indicated that IV iron treatment promotes oxidative stress, atherosclerotic plaque development, infection, hypersensitivity responses and increased cardiovascular mortality [113,115]. Studies involving apolipoprotein E (ApoE) knockout mice have highlighted that elevated iron does not cause atherosclerotic plaque progression, whereas other studies have shown that IV iron sucrose increases superoxide production and monocyte adhesion to the endothelium, instigating atherosclerotic plaque formation [116,117]. IV iron has been found to be effective for functional iron-deficiency anemia in CKD patients with high inflammation but had negative consequences on markers of oxidative stress that could have clinical implications [111]. Moreover, different preparations of IV iron carry different risks. Iron dextran carries a higher risk of adverse reactions, including type 1 hypersensitivity reactions, in comparison to iron sucrose, sodium ferric gluconate and ferric carboxymaltose [118,119]. The recommended adult doses of iron sucrose and sodium ferric gluconate carry lower risks in CKD [120,121]. The Ferumoxytol for Anemia of CKD Trial (FACT), a randomized, phase 4 study [122], reported comparable efficacy and safety of ferumoxytol and iron sucrose in patients with CKD undergoing hemodialysis. However, as clinical trials such as the ferinject assessment in patients with iron-deficiency anemia and non-dialysis-dependent chronic kidney disease (FIND-CKD) and a randomized trial to evaluate intravenous and oral iron in chronic kidney disease (REVOKE) were un-unanimous in their conclusions on IV iron safety, its use remains a subject of continuous debate [112,114]. However, the use of IV iron should be with caution since iron overload has been detected by MRI in hemodialysis patients with relatively low serum ferritin levels [123], suggesting that iron overload can occur in CKD patients receiving standard doses of IV iron. Thus, it was recommended that the dose of IV iron should be reduced to <250 mg/month to avoid iron overload in CKD patients [124]. Recommendations on iron management in CKD patient care are an ‘ongoing process’ because of limited research evidence. The outcomes of several randomized controlled trials (RCTs) and observational studies are varied regarding the effectiveness and adverse effects of iron or ESA supplementation. Heterogeneity of confounders have been associated with the study design and can be due to the type, dosage, duration or route of iron administration, population size and the inherent variability within the baseline [125,126] hematological profile of patients.

## 12. Novel Therapies for the Treatment of Anemia of CKD

Although recent therapies offer benefits to most patients, some patients remain anemic and, therefore, there is a drive to develop novel therapies to address persistent anemic conditions (Table 2).

### 12.1. Targeting Hepcidin

High levels of hepcidin recorded in CKD patients act to impair absorption and mobilization of iron.

Furthermore, the chronic inflammation which manifests in CKD patients results in the production of pro-inflammatory cytokines including interleukin-6 (IL-6), which has been shown to stimulate the synthesis of hepcidin. Currently, inhibitors of hepcidin production are being investigated. The two main pathways involved in regulating hepcidin expression are the BMP6-HJV-SMAD and the IL-6-STAT3 signalling pathways. Studies have revealed the existence of a cross-talk between the two pathways. In vitro studies have shown that therapies which act to inhibit the BMP pathway by sequestering ligands for the BMP receptor or antagonizing the BMP receptor also inhibit hepcidin expression via the inflammatory IL-6-STAT3 signaling pathway. Inhibitors of this pathway currently being investigated include anti-IL-6 antibodies such as Tocilizumab and IL-6 monoclonal antibodies such as Sultuximab. The safety of using these drugs needs to be verified as sultuximab, despite causing an increase in hemoglobin levels, has been associated with increased infection risk [127]. Dorsomorphin is an inhibitor of the BMP type I serine threonine kinase receptors and targets HJV and IL-6 and thus also dampens down the inflammation-induced expression of hepcidin. However, Dorsomorphin is non-selective and also inhibits the action of AMP kinase. Nonetheless, this highlights the potential therapeutic benefit of developing BMP inhibitors that can have a dual action on both BMP- and IL-6-mediated hepcidin expression [128]. Other potential antagonists or suppressors of hepcidin expression include Atorvastatin, TNFα and TGFɣ inhibitors. A 6-month administration of Atorvastatin to CKD patients in a randomized double-blind crossover study revealed a significant decrease in serum hepcidin [129]. This was concomitant with improved haematological parameters. Similarly, Sotatercept and Luspatercept are recombinant soluble activin type-II receptor-IgG-Fc fusion proteins that were reported to increase red blood cell numbers and hemoglobin levels in humans treated for renal anemia [119,130]. An anti-inflammatory Pentoxifylline (PTX)—a phosphodiesterase inhibitor of anti-TNF-alpha activity—has also been proposed as a potential therapy for different disorders, including anemia, and its use in CKD awaits further research [131].

### 12.2. Hepcidin-Ferroportin Axis

Currently under investigation are newer potential therapies that will target the hepcidin–ferroportin axis in the treatment of anemia in CKD. This axis can be targeted at various points. For example, direct hepcidin antagonists, such as hepcidin antibodies, are currently in clinical trials and are thought to inhibit the action of hepcidin. These antibodies have been shown to bind both human and monkey hepcidin and inhibit its action on ferroportin, and as such, enhance the absorption of dietary iron and promote its mobilization from iron stores for use in erythropoiesis [132]. An additional direct hepcidin antagonist currently under development is hepcidin RNA interference (RNAi), which is predicted to inhibit hepcidin gene expression, to promote FPN function and thereby elevate iron levels [133].

Some therapies are also exploring FPN stabilizers, which make FPN less sensitive to the action of hepcidin, thereby promoting elevated, optimal iron efflux into circulation. These stabilizers tend to reduce hepcidin expression and inhibit its action while preventing FPN degradation, which aid in the treatment of absolute iron deficiency in anemia of CKD. One example of such an FPN stabilizer is the anti-ferroportin monoclonal antibody, which prevents the interaction between hepcidin and FPN [128]. Two other monoclonal antibodies, LY3113593 and LY2928057, targeting BMP6 and ferroportin, respectively, tested in CKD patients resulted in an increase in haemoglobin and reduction in ferritin (compared to the placebo) [134]. Serum iron efflux increased through LY2928057 binding to ferroportin and blocking interactions with hepcidin. In the same vein, LY3113593 blocked BMP6 binding to its receptor to decrease hepcidin expression.

### 12.3. Targeting Hif1α Inhibitors

The stabilization of HIF via a prolyl hydroxylase inhibitor (HIFα-PHI) system is a novel approach that may also be an effective therapeutic target in the treatment of anemia of CKD as EPO deficiency contributes greatly to this condition. HIF1α regulates renal EPO production and erythropoiesis (described in Section 9). Thus, this approach involves manipulating a physiological regulatory process rather than the conventional EPO administration. Examples of HIF-PHIs that are in clinical trials include Vadustat, Daprodustat and Roxadustat [97,135,136,137,138]. Phase II clinical trials of HIF1α stabilizers have concluded the effectiveness and safety for short-term use [128,139]. More recently, a Phase II, randomized, double-blind, placebo-controlled, trial showed a dose-dependent increase in Hb compared to placebo in adult CKD patients with anemia after 6 weeks of Desidustat (ZYAN1) treatment [140]. Desidustat (ZYAN1) is an oral hypoxia-inducible factor prolyl hydroxylase inhibitor (HIF-PHI) that stimulates erythropoiesis. Another novel hypoxia-inducible factor prolyl hydroxylase inhibitor, Molidustat, has the potential to treat anemia of CKD by increasing erythropoietin production and improving iron availability particularly, in non-dialysis patients [141]. However, HIFs have roles in other biological pathways, including in the expression of vascular endothelial growth factor (VEGF), which is associated with retinal disease and cancer [142]. Consequently, the long-term safety of HIF-PHIs, and possibly Hif 2α, needs to be elucidated, in particular, for long-term therapy [143].

### 12.4. Other Compounds

An additional novel therapeutic strategy in the treatment of anemia in CKD is the development of engineered lipocalins called anticalins, which are able to bind small hydrophobic molecules such as hepcidin and thereby inhibit it from carrying out its function [144]. Anticalin PRS-080#22 has also been shown to sequester hepcidin in a Phase I clinical trial [145]. PRS-080#22 decreased hepcidin and increased serum iron and transferrin saturation in a dose-dependent manner. In mice and in patients with deep vein thrombosis (DVT), administration of the anticoagulant, heparin, caused decreased hepcidin levels and increased mobilization of iron from splenic stores, thereby increasing the circulating iron level [145].

Heparins have been shown to be inhibitors of hepcidin expression in vitro and in vivo [146]. They suppress hepcidin expression via the BMP6/SMAD pathway and are, therefore, promising for the treatment of anemia of CKD that is, in part, exacerbated by high hepcidin levels in the patients [147]. The mechanism by which heparin antagonizes the BMP/SMAD pathways awaits future clarification.

Vitamin D has also been found to reduce hepcidin gene transcription, lower serum levels by 50% in healthy individuals within 24 h, enhance erythropoiesis and reduce inflammation [135,148]. Moreover, in early-stage chronic kidney disease patients, vitamin D3 supplementation decreased hepcidin level after three months of administration [149]. However, the calcitriol form of vitamin D did not reduce serum hepcidin concentrations among individuals with mild to moderate CKD [150]. Similarly, in pregnant women, vitamin D3 supplementation did not influence hepcidin, ferritin, or inflammatory status, indicating no beneficial effect in alleviating iron depletion in the subjects [151]. Further studies are needed to confirm the long-term effect of vitamin D in CKD patients.

#### Potential New Therapy for Anemia of Chronic Disease in the Future

Recently, the bone-secreted hormone fibroblast growth factor (FGF23) inhibitor has been advocated for the treatment of anemia in CKD patients. FGF23, apart from its canonical functions in bone mineralization for the regulation of phosphate vitamin D homoeostasis exerts a pleiotropic role in iron metabolism in CKD patients [152]. Iron deficiency, inflammation and EPO have been shown to increase FGF23 protein levels and cleavage [153,154,155], resulting in an increase in the anemic condition in CKD. Conversely, studies have also shown that FGF23 may promote anemia, iron deficiency, and systemic inflammation, particularly in CKD [142,156]. Hence, inhibitors of FGF23 could be employed to stimulate erythropoiesis and treat anemia in CKD patients. Agoro and others [157] reversed anemia and iron deficiency in a mouse model of CKD by inhibiting and blocking the FGF23 signalling pathway with its peptide antagonist. The mice displayed increased erythropoiesis, serum and ferritin levels and reduced erythroid apoptosis and inflammation. Table 2 summarizes the potential novel therapies for anemia in CKD.

## 13. Conclusions

Is anemia a symptom, disorder or disease? Iron deficiency occurs insidiously as a symptom or syndrome over a spectrum of severity, with low hemoglobin as a later manifestation of extreme deficiency [11]. Absolute anemia, that manifests after the depletion of iron stores, is a clinical disease condition. However, functional iron-deficiency anemia is a risk factor for several ailments, disorders and diseases. In general, the etiology of anemia is multifactorial and this necessitates diverse therapeutic guidelines in the management of the syndrome. Hence, there are variations in guidelines specifications or consensus statements for the therapy of different stages of iron deficiency and anemia in different disorders. When dietary iron sources are limiting, iron formulations are prescribed as oral supplements. Parenteral or intravenous iron therapy becomes the choice of therapy for iron-deficiency anemia for these patients intolerant or refractory to oral iron administration. However, recommendations to start therapy vary with different conditions. For example, for IBS, CKD or anemia of heart failure, the recommendation to commence therapy is based on a wide range of serum ferritin levels (30–299 ng/mL) and when transferrin saturation is below 20%. A key regulator of iron homeostasis is hepcidin, which contributes to anemia by reducing the absorption of iron from the diet, as well as through diminishing the mobilization of iron from iron stores. One of such anemia is that associated with CKD, which manifests absolute iron deficiency and iron-restricted functional anemia and impaired erythropoiesis. Current therapy is successful in some patients in alleviating the signs and symptoms of anemia such as weakness, headache, vertigo and fatigue; however, despite current intervention, the disorder remains endemic in patients. The current review describes several novel therapies to tackle this devastating condition and correct elevated hepcidin levels in CKD patients not responding to current interventions. However, the efficacy, tolerability and side effect profiles of these novel therapies in CKD patients have not been fully elucidated. It is encouraging that studies are on-going on some of these novel therapeutic approaches that can be translated into clinical applications.

## Figures and Tables

**Table 1:** Features of anemia of inflammation [17].

Cells/Tissue	Cytokine	Effector Function
Hepatocytes	IL6 and lipopolysaccharide (LPS)	Induction of hepcidin expression in the liver. Hepcidin inhibits iron efflux from the macrophages and the duodenum by blocking or degrading ferroportin.
Macrophage		
	Lipopolysaccharide (LPS) and TNFα	Increase DMT1 expression and uptake of ferrous iron(Fe^2+^).
	TNFα	Promotes damage of erythrocyte membranes and the stimulation of phagocytosis.
	IFNɣ and LPS	Decrease expression of ferroportin to inhibit iron efflux and accentuated by hepcidin.
	TNFα-IL1, IL6 and IL10	Induction of ferritin expression, storage and retention of iron within macrophages.
Monocytes	IL10	Enhances TfR1 expression to promote uptake of transferrin-bound iron.
Kidney	TNFα, IFNɣ and IL1	Dysregulated erythropoietin receptor EPOR expression and signalling via blunted expression of Scribble (Scb) and inhibition of erythropoietin (EPO) and erythroferrone (ERFE) which production. The cytokines also directly inhibit the differentiation and proliferation of erythroid progenitor cells.

TNFα, tumor necrosis factorα; TfR1: Erythropoietin receptor 1; IFNɣ, interferon ɣ; DMT1, divalent metal transporter 1.

**Table 2:** Summary of potential therapies for anemia of chronic kidney disease (CKD).

Name	Mode of Action	Adverse Effects	References
Hepcidin antagonist, e.g., hepcidin antibodies RNA Interference Atorvastatin, Sotatercept Luspatercept	Inhibit hepcidin action Inhibit hepcidin expression and promote FPN function	Viral delivery system—risk of random genome integration. Unfavorable immunological responses	[121,122,132,133,144,158]
Hepcidin binding proteins Lipocalin, e.g., Anticalin (PRS-080)	Inhibit hepcidin function	Non-specificity	[159]
Hepcidin production inhibitors BMP inhibitors, e.g., soluble HJV, Dorsomorphin, Anti-BMP6 monoclonal antibody	Inhibit hepcidin expression and the BMP6-HJV-SMAD pathway	Unknown	[134,160]
Anti- IL6 monoclonal antibody, e.g., Siltuximab	Inhibits IL-6 STAT3 signaling cascade	Unknown	[158]
Heparin	Decreases hepcidin levels Increases mobilisation of iron stores	Bleeding Thrombocytopenia Hyperkalaemia Alopecia Osteoporosis	[161]
Vitamin D	Decreases hepcidin gene transcription	In excess causes nausea, vomiting, depression, weakness, and confusion.	[148,149,150]
FPN stabilizers, e.g., anti-ferroportin monoclonal antibody	Increase ferroportin action	Unknown	[130,134]
HIF-PHDI, e.g., Roxadustat Daprodustat, Vadadustatt, Molidustat,	Increase endogenous EPO expression	Pulmonary hypertension Increase in VEGF Tumour progression	[97,98,134,136,141]
Anticalin PRS-080#22	Decreases hepcidin levels Increases mobilisation of iron stores	Unknown	[144]
FGF23 inhibitor	Stimulates and promotes erythropoeisis	Unknown	[153,157]

## Abbreviations

ID	iron deficiency	IDA	Iron-deficiency anaemia
GBD	Global Burden of Disease	CKD	chronic kidney disease
ACD	Anemia of Chronic Disease	IBD	inflammatory bowel disease
IV	intravenous	EPO	erythropoietin
ESA	erythropoietin stimulating agent	rHuEPO	recombinant human erythropoietin
FPN	ferroportin	DMT1	divalent metal transporter
Dcytb	duodenal cytochrome b	MCV	mean corpuscular volume
Hb	haemoglobin	BMP	bone morphogenic protein
HJV	hemojuvelin	SMAD	Mothers Against Decapentaplegic
JAK2	Janus kinase 2	STAT 3	Signal transducer and activator of transcription 3
GDF 15	growth differentiation factor 15	PDGF-BB	platelet-derived growth factor-BB
HIF	hypoxia-inducible factor	VEGF	vascular endothelial growth factor
IL	Interleukin	FCF23	fibroblast growth factor
KDIGO	Kidney Disease: Improving Global Outcomes	CHOIR	Correction of Haemoglobin and Outcomes in Renal Insufficiency
CREATE	Cardiovascular Risk Reduction by Early Anemia Treatment	TREAT	Trial to Reduce Cardiovascular Events with Aranesp
HIF-PHDI	Hypoxia-Inducible factor Prolyl Hydroxylase Inhibitor		
